# Psychological predictors of quality of life after anterior cervical discectomy and fusion for degenerative cervical spine disease

**DOI:** 10.1038/s41598-020-70437-9

**Published:** 2020-08-07

**Authors:** Arthur Wagner, Youssef Shiban, Leonie Zeller, Kaywan Aftahy, Nicole Lange, Stefan Motov, Ann-Kathrin Joerger, Bernhard Meyer, Ehab Shiban

**Affiliations:** 1 Department of Neurosurgery, Klinikum Rechts der Isar, Technical University Munich School of Medicine, Ismaninger Str. 22, 81675 Munich, Germany; 2 Department of Clinical Psychology, Private University of Applied Sciences, Göttingen, Germany; 3 Department of Neurosurgery, Universitätsklinikum Augsburg, Augsburg, Germany

**Keywords:** Psychiatric disorders, Signs and symptoms, Disability

## Abstract

We aimed to identify independent psychological predictors of quality of life (QOL) and functional outcome after anterior cervical discectomy and fusion (ACDF) for degenerative cervical spine disease. We prospectively included patients undergoing ACDF for degenerative cervical disc herniation and stenosis. Patients completed a structured psychological assessment including the Center for Epidemiological Studies Depression Scale (ADS-K), Post-Traumatic Stress Scale-10 (PTSS-10), State Trait Anxiety Inventory-State Anxiety and - Trait Anxiety (STAI-S and STAI-T) and Anxiety Sensitivity Index-3 (ASI-3) before surgery, after 3 and 12 months. Outcome measures included EuroQol-5D (EQ), Short Form-36 (SF-36) and Oswestry Disability Index (ODI) scores. Of 104 included patients who underwent ACDF between March 2013 and November 2017, 92 completed follow-up after 3 and 12 months. The mean Visual Analogue Scale (VAS) scores for neck pain (− 1.4; *p* < .001) and arm pain (− 1.8; *p* = .031) significantly decreased by 12 months. QOL scores significantly increased by 3 months (EQ: + 0.2; *p* < .001; SF-36 PCS: + 6.2; *p* < .001; SF-36 MCS: + 2.5; *p* = .044), a benefit which was retained at 12 months. Linear regression analyses identified statistically significant predictors in preoperative ASI-3, SF-36 MCS and STAI-S for postoperative QOL and ODI scores. There is a benefit for patients in terms of quality of life and function after undergoing surgery for degenerative cervical spine disease. With the ASI-3, SF-36 MCS and STAI-S there exist some predictors for postoperative QOL and ODI scores.

## Introduction

Degenerative diseases of the cervical spine are known to encompass a variety of pathologies producing pain, disability and impaired health-related quality of life (QOL). Surgical treatment of these pathologies via an anterior cervical discectomy and fusion (ACDF) procedure has been documented with favourable results in an abundant number of case series^1–7^. Despite several testaments to the benefit of the procedure, there are patients who may benefit to a lesser extent than desired, at times requiring the operating surgeon to consider altering their treatment strategy accordingly. Various research groups have investigated somatic predictors of impaired QOL after ACDF^8–10^. However, while evidence demonstrating the significant influence of a psychological predisposition of patients on QOL and the functional outcome after surgery for degenerative diseases in the thoracolumbar region continues to emerge, to the best of our knowledge, only a few studies have addressed these aspects after ACDF prospectively^11–15^.

The available evidence concerning outcome for the cervical spine suggests somewhat conflicting results, without an unambiguous solution for a routinely feasible and reliable psychological assessment of patients. To assume that the psychological profile directly modulates a patient’s perception of pain and disability seems plausible, but a concise psychological assessment for preoperative preparation in clinical practice has not yet been established. In principle, the identification of independent psychological scores that predict the degree of clinical benefit after ACDF may facilitate the implementation of a preemptive psychosocial intervention in the future, thereby possibly optimizing the surgical outcome. Most studies with a retrospective design have reported an inverse correlation between preoperative depression scores and the postoperative QOL improvement^16–18^. One such investigation drew these conclusions from retrospective data, with a limited follow-up time of 7 months in the control group being the primary limitation^16^. Elsamadicy et al. retrospectively compared patients diagnosed with depression and treated with antidepressants at least 6 months prior to surgery with a non-depressive control group, concluding that the pretreatment resulted in similar outcomes between the groups after ACDF^18^.

In our study, standardized assessments of psychological scores and various dimensions of QOL were conducted before surgery and during follow-up. The psychological profile of patients gathered from the individual scores represented a surrogate index of the patients’ depression and anxiety in light of undergoing surgery of the cervical spine. With this set-up, we hypothesized that our psychological test battery of depression, anxiety and PTSD scores may be used for prediction of QOL increases after ACDF surgery. Our primary outcome was thus represented by the relationship between the preoperative psychological baseline scores and QOL after 12 months, as examined by multiple regression analyses.

We applied a similar methodology as studies concerning this topic previously published by our study group by prospectively screening patients scheduled for elective ACDF at our institution^19,20^. Our preoperative assessment encompassed various scales of depression and anxiety disorders, which we sought to identify via the following commonly employed psychological instruments: the *Center for Epidemiological Studies Depression Scale*^21,22^ (*Allgemeine Depressionsskala*; ADS), *Post-Traumatic Stress Scale–10*^23^ (PTSS), *State Trait Anxiety Inventory–State Anxiety* (STAI-S), *State Trait Anxiety Inventory–Trait Anxiety*^24^ (STAI-T) and *Anxiety Sensitivity Index-3*^25,26^ (ASI). The scores of the aforementioned instruments were correlated with the scores for the *EuroQol*^27^ (EQ), *Short Form 36*^28^ (SF-36) and *Oswestry Disability Index*^29,30^ (ODI).

## Methods

### Study design

This is a monocentric prospective cohort study conducted between March 2013 and November 2017 at the Department of Neurosurgery, Technical University Munich. We recruited a cohort of patients scheduled for an elective ACDF procedure involving one to three consecutive segments that were affected by a degenerative spondylotic deformity defined as stenosis by spondylophytes in the central or lateral compartments, disc herniation and degeneration, or a combination of the aforementioned conditions. The analysis included patients aged over 18 years exhibiting symptoms consistent with pathomorphologic changes found in imaging results for a minimum duration of 6 weeks. No financial compensation was provided for participating patients. Conventionally, two distinct clinical syndromes are associated with these pathomorphologic changes: most predominantly, patients with cervical spondylosis presented with neck pain, pain radiating to the upper extremities or both types of pain. In the presence of clinically manifested cervical spondylotic myelopathy (CSM), decompression was indicated and offered irrespective of the symptom duration. CSM was diagnosed by a board-certified attending neurosurgeon at our institution by an examination for gait ataxia, coordinative disorders, latent weakness of the hands, pronounced tendon reflexes of the lower extremities and clonus. In contrast, cervical radiculopathy (CR) manifested as radiating pain and possible sensory dysfunction at the dermatome of the affected nerve root. In cases of acute neurological deterioration due to CR or CSM, defined as severe neurological motor deficits with a grade of 3 or lower according to the Medical Research Council scale or vegetative symptoms, patients were not eligible for study participation after screening. The standard preoperative diagnostic work-up of the cervical spine beyond the thorough clinical examination comprised magnetic resonance imaging (MRI) scans and additional computed tomography (CT), dynamic radiography or bone densitometry assessments, as needed on a case-by-case basis.

### Psychological instruments

An assortment of psychological instruments, including the German versions of the ADS, PTSS, STAI-S, STAI-T and ASI, constituted the prime focus of this study. All of these instruments have been used extensively and validated in a multitude of explorative studies, which provided instructions and tested cut-off values^21–23,25,31–35^. For more information on the psychological scales, see Table 1. The rationale behind including the various instruments aimed at an assessment of depression, anxiety and posttraumatic stress syndrome stemmed from the design of similar published studies that employed these instruments entirely or in part^12,34,36–40^. There is conclusive evidence that depression, anxiety and posttraumatic symptoms influence surgical outcomes after neurosurgical procedures^17,36,41–44^. The neuropsychologists of our study group hence designed an assortment of psychological instruments for a comprehensive account of the patients’ psychological profile. While 4 out of the 8 sections of the SF-36 assessment focus on physical health and are summarized through the SF-36 PCS, the other 4 function as markers of emotional health and are aggregated into the SF-36 MCS.

**Table 1:** Overview of the standardized questionnaires used in the study.

Questionnaire	Description	Cut-off
General Depression Scale (Allgemeine Depressionsskala; ADS-K)^21^	This index is based on the *Center for Epidemiological Studies Depression Scale* (Radloff. 1977) and was devised to determine depression levels for outpatients. The 15 items are sensitive to dysthymic disorders, not only to major depression	≥ 18
State Trait Anxiety Inventory (STAI-T and STAI-S)^68^	This two-part questionnaire was conceived to measure the two different dimensions of anxiety with 20 items each: a stable character trait and personal disposition; a transient state as a function of current influences	> 40
Post-Traumatic Stress Scale (PTSS-10)^69^	The scale consists of 10 items that check for pathognomonic symptoms of post-traumatic stress disorder	≥ 18
Anxiety Sensitivity Index (ASI-3)^25,26^	This index is a measure of susceptibility to states of anxiety and perception of potentially hazardous symptoms. 18 items	> 30
European Quality of Life Questionnaire (EuroQol)^70^	The concept of quality of life leans on 5 dimensions of everyday life including *Mobility. Self-care. Usual Activities. Pain/Discomfort* and *Anxiety/Depression.* The respective scores are summarized into a single index on the *Visual Analogue Scale* (VAS). Higher scores on the VAS indicate better quality of life	Score 0.0–1.0
Short Form Health Survey (SF-36)^28^	With its 36 items. the SF-36 gauges 8 aspects of health-related quality of life of a patient. The aspects may be summarized in the *Physical Health Component Summary Score* (PCS) and *Mental Health Component Summary Score* (MCS). Higher values signal favourable physical & mental capacity	Score 0–100
Oswestry Disability Index (ODI)^30^	The ODI has been a reliable tool for the assessment of functional impairment in patients with degenerative spine disease. Each of the 10 items addresses certain domains of everyday life and autonomy. A score of 0–5 is assigned to each answer and multiplied by 2 with higher scores representing higher disability	Score 0–100

### Study procedures

The preoperative screening and all psychological assessments used in the follow-up sessions at 3 and 12 months after surgery were conducted by a trained neuropsychologist. The postoperative developments in health-related QOL were assessed by the *EuroQol 5D* (EQ) and *Short Form 36* (SF-36), which was further classified into the Physical and Mental Component Scores (PCS and MCS). The Oswestry Disability Index (ODI) provided an evaluation of the self-perceived functional capacity of the patients. Furthermore, we acquired the history of prior psychiatric consultations, intake of psychiatric medication and in-patient psychiatric treatment during the recent 12 months.

The intensity of pain was evaluated both by the Visual Analogue Scale (VAS), which depicts intensity on a continuous, linear scale from the least to the most intense level of pain, and the SF-36 Bodily Pain subscale. The assessments of pain according to these scales pertain to the given patient’s predominantly affected site, i.e., arm pain for an individual with CR.

A Likert-scaled “Patient Satisfaction” item spanning from 1, corresponding to “no improvement”, to 10, corresponding to “full resolution of complaints”, allowed patients to subjectively rate their individual surgical success.

ACDF surgery was conducted according to well-established and documented standards; a ventral left paramedian transversal incision at approximately the level of the pathology was made for complete discectomy as well as reduction of spondylophytes at the uncinate processes and neuroforamen bilaterally. The cartilaginous end-plates were fully ablated to allow for subsequent bony fusion. Supplemental ventral plate osteosynthesis was conducted in the presence of apparent instability in the imaging results or risk factors for non-fusion, including smoking or recent long-term steroid use.

### Statistical analyses

For the primary outcome, we performed multiple linear regression analyses with stepwise forward selection to predict the QOL scores after 12 months as dependent variables and preoperative psychological assessments as independent variables. For every dependent variable, a Q-Q plot was generated, and a normal distribution of the residuals, a prerequisite for the regression analysis, was demonstrated by these plots. Similarly, the linear relationships between each of the dependent variables and independent variables were assessed by scatter plots. The Durbin-Watson test (d) was used to detect and exclude first-order autocorrelation in each of the regression models. The composite scores of the QOL scales, the ODI and the Patient Satisfaction scale were fitted as continuous dependent variables in separate regression models, while the preoperative psychological scores were included as independent variables.

A variable was only included if the significance level of its F value was less than 0.05. The estimated unstandardized coefficient statistics, t-values and corresponding p-values were used for reporting the results.

The secondary analyses involved the developments in QOL, disability and pain scores over follow-up times and their correlation with the independent variables and demographics, which were addressed with three-level repeated measures analysis of variance (rANOVA), one-way ANOVA for multiple pairwise comparisons, Student’s t-test and the Chi-square test. The Bonferroni method was used to correct for multiple comparisons. Further, the proportions of patients with pathological depression and anxiety scores were compared over time via the Cochran Q test followed by posthoc pairwise testing with the McNemar test.

We used the 25th version of IBM SPSS for the statistical analyses, and the level of significance was defined a priori as *α* = *0.05*.

### Ethical considerations

All procedures were indicated and conducted in compliance with our department’s standards and the Declaration of Helsinki. The operating surgeons and ward personnel were blinded to study participation. The local ethics committee approved the study (Ethikkommission der Technischen Universität München, registration no. 409/13), and informed consent was obtained from all participants before study inclusion.

### Ethical approval

All procedures performed in studies involving human participants were in accordance with the ethical standards of the institutional and/or national research committee and with the 1964 Helsinki declaration and its later amendments or comparable ethical standards. The study group acquired approval by the local ethics committee (Ethikkommission der Technischen Universität München, registration no. 409/13).

### Informed consent

Every participant of this study provided written informed consent.

## Results

### Epidemiology

Between March 2013 and November 2017, 159 eligible patients with degenerative symptomatic cervical spine disease scheduled for elective ACDF were screened, and 104 (65.4%) opted for study participation. Of these patients, 92 (89.5%) completed follow-up examinations 12 months after surgery. Table 2 summarizes the baseline characteristics of this cohort, which were used in all subsequent analyses. Decompression for adjacent segment disease after a prior procedure was performed in 6.5% of the cases. A ventral plate was added in 39.1% (n = 36) of the cases, while the remaining 60.9% (n = 56) received an ACDF with a stand-alone cage interbody fusion.

**Table 2:** Baseline characteristics of cohort stratified by subgroups of different clinical presentations.

		CSM	CR	CSM + CR	Total
Number		64	19	9	92
Age in years (SD; range)		61 ± 12.4 (30–85)	57 ± 11.2 (31–77)	64 ± 11.5 (49–82)	61 ± 12.1 (30–85)
Gender	Female	57.8%	68.4%	55.6%	59.8%
Relationship status	Single	17.5%	27.8%	12.5%	19.1%
	Married	66.7%	55.6%	62.5%	64.0%
	In a relationship	6.3%	11.1%	0.0%	6.7%
	Widowed	9.5%	5.6%	25.0%	10.1%
Education level	Secondary School	56.3%	42.1%	77.8%	55.4%
	High school	43.8%	57.9%	22.2%	44.6%
Segments	1	54.0%	57.9%	22.2%	51.6%
	2	33.3%	26.3%	44.4%	33.0%
	3	12.7%	15.8%	33.3%	15.4%
Prior surgery at or adjacent to index level	No	92.2%	94.7%	100.0%	93.5%
	Yes	7.8%	5.3%	0.0%	6.5%

*CSM* cervical spondylotic myelopathy, *CR* cervical radiculopathy, *SD* standard deviation.

Most patients presented with signs and symptoms of cervical myelopathy exclusively (n = 64; 69.6%), 19 (20.7%) had radiculopathy only and 9 (9.8%) presented with a mixture of these signs and symptoms.

There were no revision surgeries due to immediate complications and no mortality during the follow-up period.

### Psychological assessment

**Figure 1:**
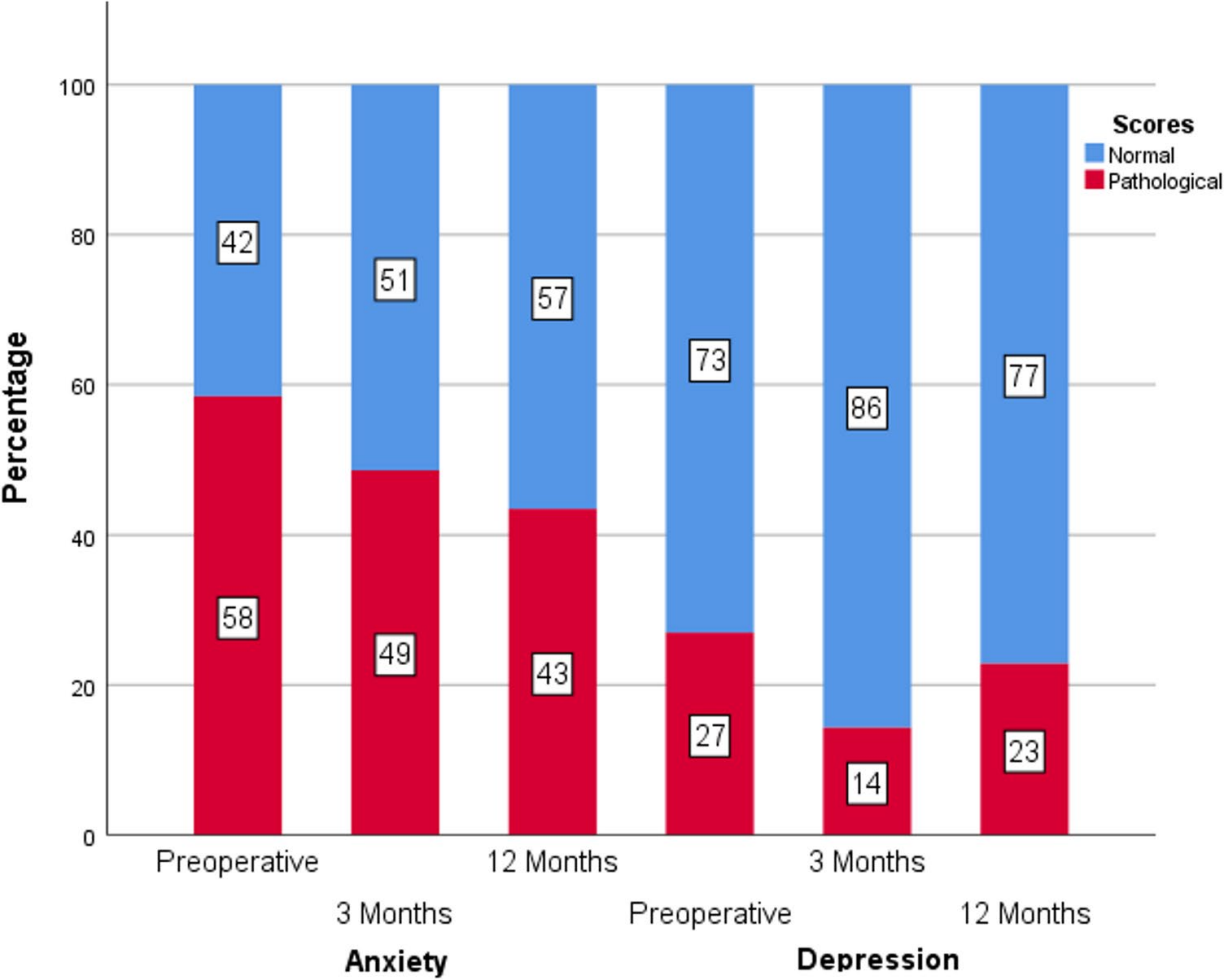
Proportion of patients with anxiety and depression scores above their cut-offs before surgery and on follow-up, respectively. Anxiety: Cochran Q *p* = .060; Depression: Cochran Q *p* = .156.

A total 20.7% of the patients stated at baseline that they received psychiatric treatment according to the above definition within the previous 12 months. The proportion of patients with pathological STAI scores reached 58% at baseline and declined to 43% after 12 months (Cochran Q: *p* = 0.060; Fig. 1). The proportion of patients with pathological depression scores decreased from 27 to 23% after 12 months, missing significant differences in the omnibus testing (Cochran Q: *p* = 0.156; Fig. 1).

**Figure 2:**
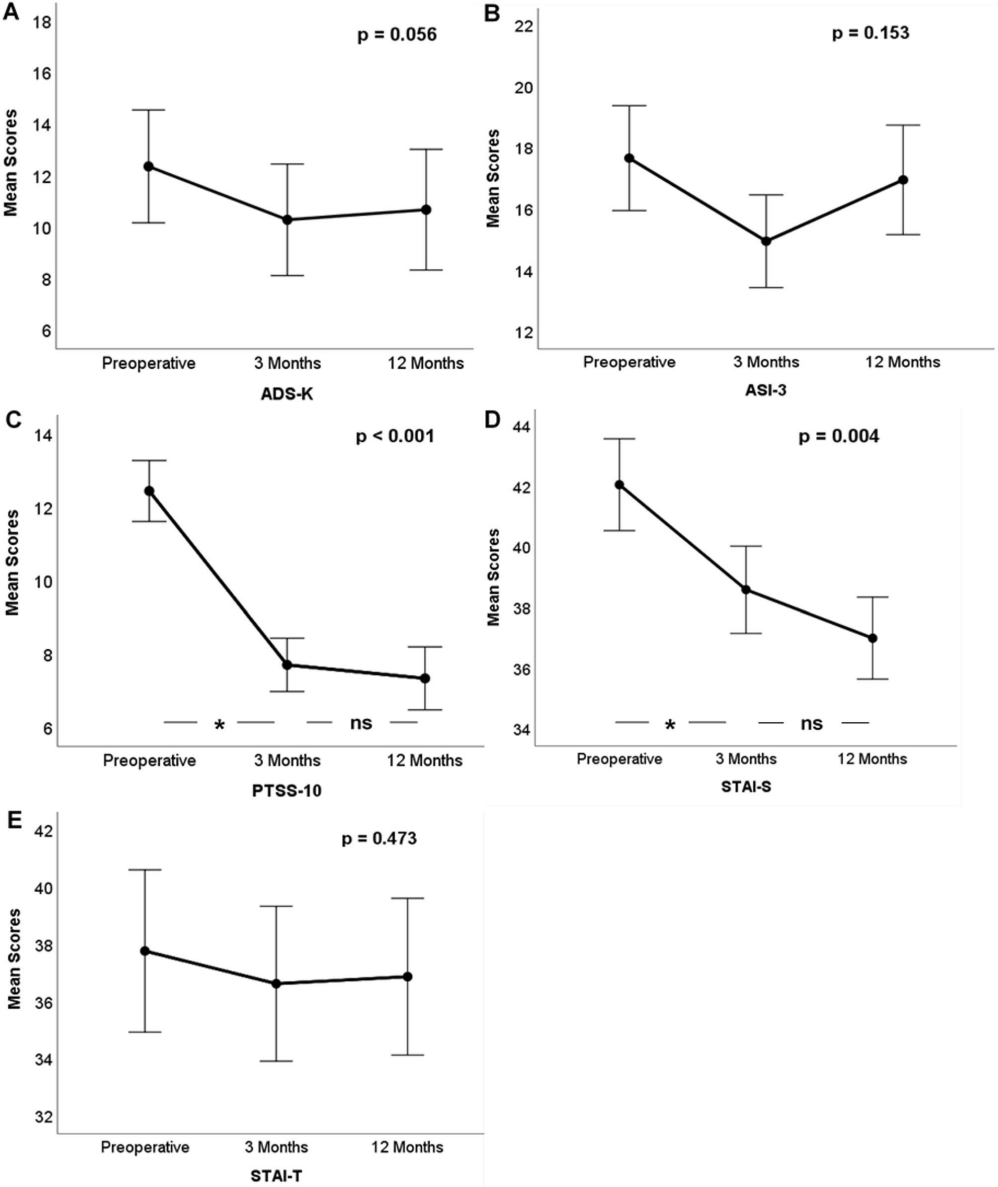
Development of mean scores with 95% confidence intervals of ADS-K **(A)**, ASI-3 **(B)**, PTSS-10 **(C)**, STAI-S **(D)** and STAI-T **(E)** from baseline to follow-up after 12 months. P—overall *p*-values calculated by one-way rANOVAs; asterisks denote significant mean differences in pairwise comparisons; *ns* non-significant.

Between baseline and 12 months, the mean scores of the PTSS (*F*(2,90) = 35.216; *p* < 0.001; *η*^2^ = 0.345) as well as the STAI-S (*F*(2,90) = 6.536; *p* = 0.004; *η*^2^ = 0.088) decreased significantly for both scales (Fig. 2). The numerical decline of mean ADS scores missed statistical significance over the 12 months of follow-up (*F*(2,90) = 3.038; *p* = 0.056; *η*^2^ = 0.043), although reaching a significant difference of means between baseline and 3 months (*F*(2,90) = 5.266; *p* = 0.025; *η*^2^ = 0.072; Fig. 2). Neither ASI-3 (*F*(2,90) = 1.984; *p* = 0.153; *η*^2^ = 0.028) nor STAI-T (*F*(2,90) = 0.726; *p* = 0.473; *η*^2^ = 0.011) had significant differences in the repeated measures ANOVA.

### Pain, disability and quality of life

Mean pain intensity at baseline amounted to a score of 6.0 (*SD* = 2.1) for neck pain and 5.6 (*SD* = 2.5) for arm pain, as measured by a VAS from 0 to 10 across the entire cohort. Similarly, the mean Bodily Pain item scores on the SF-36 were 32.8 (*SD* = 23.5) for neck pain and 34.8 (*SD* = 23.3) for arm pain. For neck pain, both the SF-36 Bodily Pain subscale (*t*(90) = − 2.663; *p* < 0.001) and VAS (*t*(90) = -5.463; *p* < 0.001) parameters improved significantly by 12 months (Table 3). Likewise, arm pain improved significantly on the SF-36 subscale (*t*(90) = − 1.893; *p* = 0.044) and VAS (*t*(90) = − 2.017; *p* = 0.031) after 12 months.

**Table 3:** Mean scores and standard deviations of pain intensity as measured by the SF-36 Bodily Pain subscale and the VAS scale at baseline, after 3 and 12 months, stratified by pain location.

	Neck pain	Arm pain
SF-36 bodily pain preoperative (SD)	32.8 ± 23.5	34.8 ± 23.3
SF-36 bodily pain at 3 months (SD)	49.2 ± 26.0	55.7 ± 20.9
SF-36 bodily pain at 12 months (SD)	50.3 ± 24.6	51.1 ± 21.3
VAS pain preoperative (SD)	6.0 ± 2.1	5.6 ± 2.5
VAS pain at 3 months (SD)	4.4 ± 2.4	3.7 ± 2.3
VAS pain at 12 months (SD)	4.5 ± 2.2	3.9 ± 2.1
**Changes after 12 months**		
SF-36 bodily pain (SD)	17.5 ± 26.1	16.3 ± 32.8
Intragroup comparison, P	< .001	.044
VAS pain (SD)	−1.4 ± 2.3	−1.8 ± 3.2
Intragroup comparison, P	< .001	.031

P—two-sided pairwise comparisons by paired t-tests *p*-value.

*SD* standard deviation.

**Figure 3:**
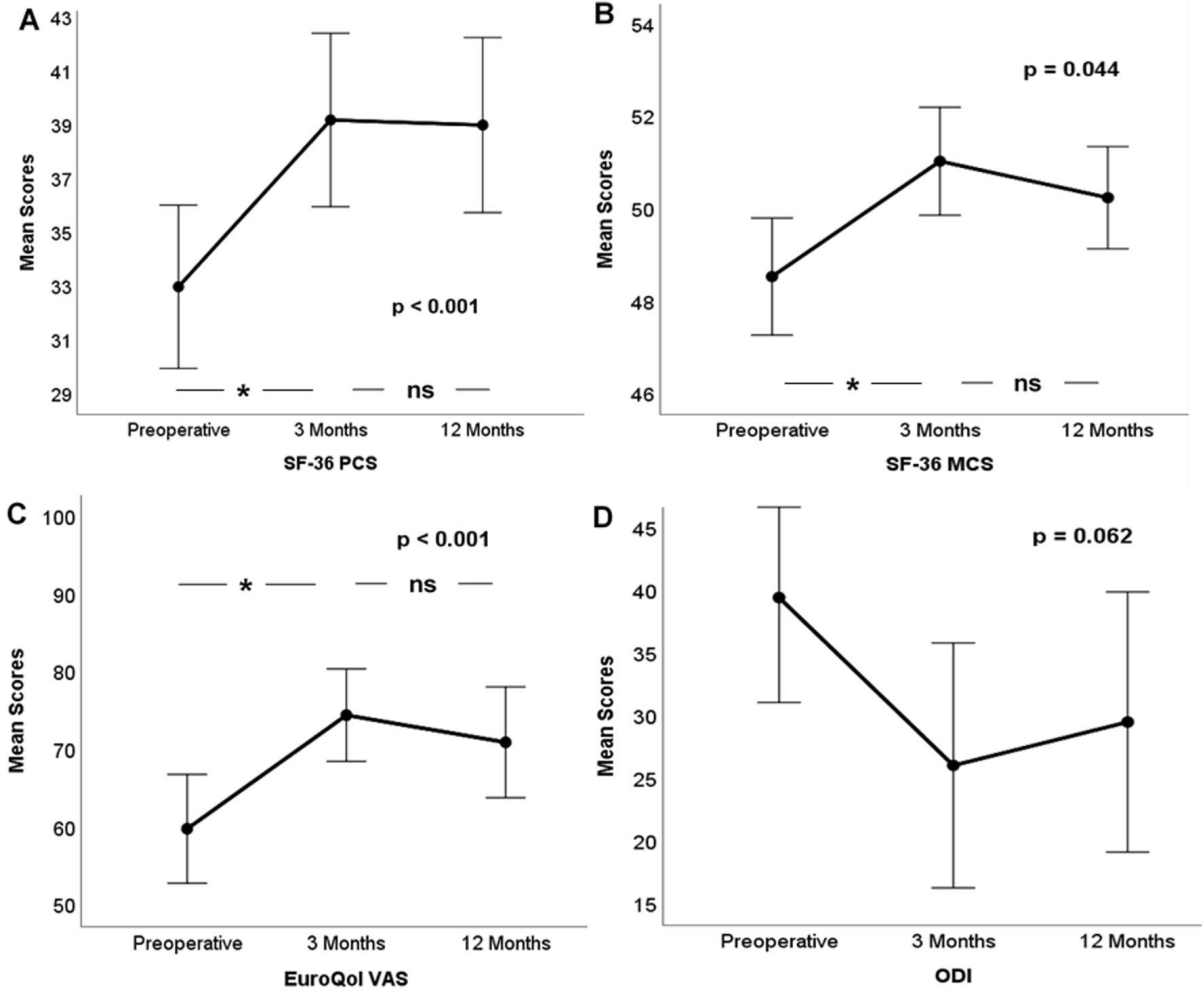
Development of mean scores with 95% confidence intervals of *SF-36 Physical Component Scale* (SF-36 PCS; **A**), *SF-36 Mental Component Scale* (SF-36 MCS; **B**), *EuroQol Visual Analogue Scale* (EuroQol VAS; **C**) and *Oswestry Disability Index* (ODI; **D**) from baseline to 12 months after surgery. P—overall *p*-values calculated by one-way rANOVAs; asterisks denote significant mean differences in pairwise comparisons; *ns* non-significant.

In the rmANOVA, the QOL scales SF-36 PCS (+ 6.2; *F*(2,90) = 15.348; *p* < 0.001; *η*^*2*^ = 0.231), SF-36 MCS (+ 2.5; *F*(2,90) = 5.360; *p* = 0.044; *η*^*2*^ = 0.092) and EQ VAS (+ 0.15; *F*(2,90) = 12.629; *p* < 0.001; *η*^*2*^ = 0.179) all exhibited significant improvements over time, while the disability scale ODI did not (+ 3.5; *F*(2,88) = 3.129; *p* = 0.060; *η*^*2*^ = 0.207; Fig. 3).

Posthoc pairwise t-tests with Bonferroni corrections for multiple comparisons revealed significant differences between the mean SF-36 PCS at baseline and 3 months (t(90) = − 4.651; *p* < 0.001), at baseline and 12 months (t(90) = − 5.029; *p* < 0.001), but not between 3 and 12 months (t(90) = − 0.009; *p* = 0.993). For the mean SF-36 MCS scores, a significant difference was found between baseline and 3 months (t(90) = − 2.315; *p* = 0.025), but not between baseline and 12 months (t(90) = − 0.907; *p* = 0.234) or between 3 and 12 months (t(90) = 0.835; *p* = 0.407). For EQ VAS, significant differences were found between baseline and 3 months (t(90) = − 4.330; *p* < 0.001) as well as between baseline and 12 months (t(90) = − 3.523; *p* = 0.001), but not between both follow-ups (t(90) = 1.447; *p* = 0.153).

A satisfactory improvement of preoperative complaints after 12 months, defined as an 8 to 10 on the ten-point Patient Satisfaction scale, was noted by 48.3% of the patients. Conversely, minor to no improvement at all was noted by 21.3% of the cohort and predefined as a 1 to 3 on the same scale. When the patients were stratified by subgroups, 44.3% of the patients presenting with CSM and 42.1% of those with CR subjectively improved without a significant difference between the subgroups (Chi-square: *p* = 0.127); however, a failure to improve was rated significantly less frequently by the CR subgroup (15.8%) than by the CSM subgroup (21.3%; McNemar: *p* = 0.010).

### Regression analyses of the outcome predictors

The results of the multiple regression analyses of dependent parameters are depicted in Table 4. The preoperative scores of the psychological instruments (ASI-3, ADS-K, PTSS-10, STAI-S and STAI-T) as well as the SF-36 MCS subscale were employed as predictors. The model summaries revealed that the regressions for the EQ VAS increase (*R*^*2*^ = 0.359; *F* = 2.083; *d* = 2.202; *p* = 0.029) and SF-36 PCS increase (*R*^*2*^ = 0.821; *F* = 16.017; *d* = 1.989; *p* < 0.001) predicted the dependent variables fairly well, respectively, although with a low R^2^ for EQ VAS. The models for the ODI (*R*^*2*^ = 0.447; *F* = 0.810; *d* = 1.728; *p* = 0.598) and Patient Satisfaction scale changes (*R*^*2*^ = 0.237; *F* = 1.059; *d* = 1.734; *p* = 0.416) proved to be insufficiently fit for the data, however (Table 4). For the increase in EQ VAS, the preoperative SF-36 MCS (*T* = 1.893; *p* = 0.016) and ASI-3 (*T* = − 1.279; *p* = 0.008) added significantly to the prediction model. For the increase in SF-36 PCS, the preoperative SF-36 MCS (*T* = 2.463; *p* = 0.005), ASI-3 (*T* = − 1.534; *p* = 0.042) and STAI-S (*T* = 2.406; *p* = 0.008) added significantly to the predictive model. For the decrease in ODI scores, both the preoperative SF-36 MCS (*T* = − 1.498; *p* = 0.037) and STAI-S (*T* = − 2.189; *p* = 0.034; Table 4) contributed significantly. Finally, none of the preoperative independent variables were identified as significant predictors to the Patient Satisfaction scale (Table 4).

**Table 4:** Multiple linear regression analyses using stepwise forward progression for QOL and disability scores with independent psychological predictors.

Score	EQ VAS increase			SF-36 PCS increase			ODI decrease			Patient satisfaction		
	*Β*	*T*	*p*	*β*	*T*	*p*	*β*	*T*	*p*	*β*	*T*	*p*
Preop. SF-36 MCS	**0.011**	**1.893**	**.016**	**0.453**	**2.463**	**.005**	**0.230**	**−1.467**	**.037**	−0.127	0.844	.214
Preop. ADS-K	−0.024	−0.110	.724	−4.383	−0.856	.202	−0.323	1.282	.694	+ 0.012	0.114	.856
Preop. ASI-3	**−0.054**	**−1.279**	**.010**	**−6.870**	**−1.534**	**.042**	−0.936	−0.455	.284	+ 0.515	−1.004	.495
Preop. PTSS-10	0.029	2.134	.087	1.664	0.095	.687	−1.777	0.113	.093	−0.008	0.726	.989
Preop. STAI-S	0.045	−0.092	.479	**−0.328**	**−2.406**	**.008**	**0.351**	**−2.189**	**.015**	+ 0.780	−0.694	.314
Preop. STAI-T	0.034	−0.291	.567	−0.274	0.961	.909	0.937	−0.743	.378	+ 0.240	0.089	.766
Model summary: *R*^*2*^/*F*/*p*/*d*	.359/2.083/.029/2.202			.821/16.017 / < .001/1.989			.447/0.810 /.598/1.728			.237/1.059/.416/1.734		

Positive and negative coefficients denoted by plus and minus signs, respectively. Bolded type denotes statistically significant values.

*Pp*-value, *β* regression coefficient, *T* t-statistics value, *R²*/*F*/*p*/d statistics of multiple linear regression model summary.

Additionally, a univariate correlation analysis revealed a significant, positive, and moderate correlation between pain relief after 12 months and improvement in the QOL scales (*EQ/Pain* Pearson *r* = 0.43, *p* < 0.001; *SF-36 PCS/Pain* Pearson *r* = 0.57, *p* < 0.001). There was no significant correlation between the ODI decrease and pain relief (Pearson *r* = 0.41, *p* = 0.105). The correlation between the baseline pain intensity and pain level by 12 months was negative and significant (Pearson *r* = − 0.58, *p* < 0.001), with patients suffering from a high baseline pain intensity experiencing significantly more pain relief after 12 months.

## Discussion

We conducted a prospective study to investigate psychological scores as predictors of QOL after ACDF for degenerative cervical spine disease. The assessments of psychological scores were surrogate parameters for the concept of the psychological distress of patients who undergo a surgical procedure. The results of our study showed marked improvements in all primary outcome scores except the ODI. The EQ and SF-36 PCS demonstrated statistically significant increases after 3 months, retaining this improvement after 12 months. The ODI was found to not improve significantly over the course of follow-up.

In the regression analyses, the baseline scores of the SF-36 MCS and ASI-3 both significantly contributed to the prediction models of EQ and SF-36 PCS improvements after surgery, which represented our principal goal of investigation. In addition, we identified the preoperative STAI-S score as a predictor for both the improvements of SF-36 PCS and ODI, signifying a propensity for the self-reported physical capacity of patients. In our previous investigation on patients undergoing elective surgery for the lumbar spine, the SF-36 MCS scores likewise proved to significantly predict EQ, SF-36 PCS, ODI and Patient Satisfaction scores, while the STAI-S and ASI-3 were not found to contribute to the prediction models ^20^. This inconsistency may in part be due to a rather heterogenous cohort of lumbar spine patients undergoing a broad range of procedures of varying invasivity.

For any surgical indication, the complexity of a patients’ preoperative complaints, the given pathomorphological cause and psychological distress have to be considered in advance. However, a comprehensive account of the psychological impact has so far been neglected in clinical practice, even though it arguably represents a considerable factor similar to somatic variables^1,45–49^.

The implications of the psychological scores determined prior to an elective spine procedure on postoperative success are poorly understood. What can generally be gleaned from the available studies is that despite the absence of a consistent statistical predictor, there is sufficient evidence supporting several underlying correlations that warrant routine preoperative psychological assessments. More specifically, the preoperative ASI and STAI-T scores may be interpreted to mirror the patient’s psychological capacity to withstand distress connected with a potentially life changing event, such as surgery^50,51^. This conclusion may not hold true for the STAI-S, which is aimed at depicting a temporary state of anxiety—the drastic relief of this preoperatively aggravated state of anxiety may contribute to the increase of QOL scores after surgery. These assumptions are hampered by the fact that the regression models for the ODI and Patient Satisfaction scales were generally crudely predicted by the data, with only the SF-36 PCS model having explained a fair 82.1% of variability in the data.

Despite the existence of numerous studies documenting QOL and functional outcomes after ACDF and a growing body of literature examining psychological scores in the context of lumbar spine surgery, there is considerably less evidence for the correlation between anxiety as well as depression and QOL outcomes after ACDF^13–18,39,52–57^.

The verdict seems ambiguous; studies have reported some degree of influence exerted by the preoperative psychological profile, while others failed to identify any interaction. In a prospectively conducted study by Engquist et al., a multivariate analysis of modifiers of the outcome in terms of pain and the Neck Disability Index (NDI) scores after ACDF suggested that high levels of anxiety and low EQ scores negatively impact these postoperative scores. The authors posited that the low baseline EQ scores rather than the preoperative pain level predicted greater pain relief after surgery. Another study suggested a similar conclusion, but no direct regression analysis between the somatic outcome and preoperative depression scores was provided^13^.

Likewise, Poorman et al. reported analogous results for *depressed* and *non-depressed* patients after ACDF, even though the authors found substantially decreased QOL in depressed patients prior to the surgery. Their findings, however, hinge solely on the EQ-5D sub-scale of emotional assessment, since the authors did not use any psychometric instruments for depression or anxiety^39^.

The aforementioned results imply that patients should be consistently screened preoperatively for abnormalities in their psychological profile, identifying predictors of impaired outcome after surgery; however, we see one particular fallacy with this approach. It must be emphasized that the psychological distress that accompanies somatic stressors such as pain and disability in the presence of a surgically curable condition is not sufficient evidence to disqualify a surgical candidate—on the contrary, these distressed patients may even benefit distinctly in both somatic and psychological aspects, which can provide even more incentive to proceed with the surgery. Consequently, a routine preoperative assessment may be warranted, but must be interpreted as a means to delineate variations of postoperative benefits, which are generally favourable after ACDF surgery for degenerative cervical disease, as has been described before^8,54,55,58,59^. This concept is tied to the intricate interaction between somatic symptoms and psychological distress for degenerative spine disease, that is purported in some investigations^44,60–62^. While it is difficult to definitively specify whether somatic symptoms caused psychological distress or a manifest psychiatric disease produced somatization for each individual case, we maintain that this question remains subordinate in view of parallelly improving psychological and QOL scores.

Based on these propositions and the low-risk practicability of such an assessment, an argument may be made to routinely conduct screenings of the psychological profile of patients, aiding clinicians in their perioperative consultation and management. An extrapolation of this approach sees patients undergo pretreatment by educatory measures or psychotherapy before proceeding with the surgery. In a review by Burgess et al., 11 studies with a preoperative intervention were examined, although only one was considered of high methodological quality^63^. The nature of the preoperative intervention varied considerably from a months long structured education and prehabilitation to handing out of an instruction booklet on the day of surgery. Despite no impact on postoperative QOL, return to work or postoperative complications, limited evidence demonstrated a positive influence on postoperative pain and disability scores. In an exemplary interventional study by Adogwa et al., the effect of *pretreating* a manifested anxiety disorder prior to ACDF surgery was examined. Of 27 patients, 11 patients received psychotherapy and pharmacotherapy 6 months prior to the procedure until the last follow-up, resulting in drastically and significantly increased pain relief compared to that in the control group after 6 months and thereafter. No other patient-reported outcome that reflected QOL had significantly changed, albeit the results may have been influenced by the small sample size and non-randomized nature of the study^12^. In summary, several investigations examined similar aspects of postoperative QOL after surgery for cervical spine disease, although the chosen psychological test instruments were invariably fewer in number and more selective in comparison to our rather broad assessment. Still, the identification of the SF-36 MCS as a reliable predictor in particular is in accordance to our own results. Moreso, we are able to present one of the largest comprehensively examined patient cohorts in literature.

Notably, this study succeeded in identifying predictors of QOL improvements after ACDF surgery, but failed to delineate one explicit cut-off to identify the patients at risk for failure to improve. The SF-36 MCS predictor has been prominently featured in several other publications assessing the psychological profile in the setting of cervical and lumbar spine surgery, while the ASI-3 and STAI-S have not been identified before^12,14,64–66^.

## Study limitations

The study was conducted to identify independent variables of the psychological profiles of patients that significantly influence the postoperative outcome, but no control group or additional assessments were included. Thus, observation bias may exist, as the psychological assessment itself may have induced abnormal psychological scores. Confirming the existence of this bias is a matter of debate in many works of literature and may exceed the scope of this study. In addition, the results must be interpreted in light of the high correlation between the EQ and SF-36 instruments^67^.

These limitations are magnified by the overall low number of cases in relation to the high number of statistical comparisons we employed. To address this, statistical analyses for the primary outcome were focused on the psychological parameters as tested by our assortment of instruments preoperatively, in turn neglecting independent surgical variables such as age, medication and the number of fused segments. These shortcomings would be compensated by a higher case number in a multicentric investigation.

In summary, our results merely offer correlative evidence between self-reported psychological determinants and surgical outcome.

## Conclusion

There is a benefit after ACDF for degenerative cervical spine disease in terms of quality of life. The preoperative assessment of SF-36 MCS, ASI-3 and STAI-S scores serves to predict postoperative QOL increases.

## Data Availability

The datasets generated during and/or analysed during the current study are available from the corresponding author on reasonable request.
